# Inhibitors of histone deacetylase 1 reverse the immune evasion phenotype to enhance T-cell mediated lysis of prostate and breast carcinoma cells

**DOI:** 10.18632/oncotarget.7180

**Published:** 2016-02-03

**Authors:** Sofia R. Gameiro, Anthony S. Malamas, Kwong Y. Tsang, Soldano Ferrone, James W. Hodge

**Affiliations:** ^1^ Laboratory of Tumor Immunology and Biology, Center for Cancer Research, National Cancer Institute, National Institutes of Health, Bethesda, Maryland, USA; ^2^ Department of Surgery, Massachusetts General Hospital, Boston, Massachusetts, USA

**Keywords:** vorinostat, entinostat, immunogenic modulation, antigen-processing machinery, histone deacetylases

## Abstract

The clinical promise of cancer immunotherapy relies on the premise that the immune system can recognize and eliminate tumor cells identified as non-self. However, tumors can evade host immune surveillance through multiple mechanisms, including epigenetic silencing of genes involved in antigen processing and immune recognition. Hence, there is an unmet clinical need to develop effective therapeutic strategies that can restore tumor immune recognition when combined with immunotherapy, such as immune checkpoint blockade and therapeutic cancer vaccines. We sought to examine the potential of clinically relevant exposure of prostate and breast human carcinoma cells to histone deacetylase (HDAC) inhibitors to reverse tumor immune escape to T-cell mediated lysis. Here we demonstrate that prostate (LNCAP) and breast (MDA-MB-231) carcinoma cells are more sensitive to T-cell mediated lysis *in vitro* after clinically relevant exposure to epigenetic therapy with either the pan-HDAC inhibitor vorinostat or the class I HDAC inhibitor entinostat. This pattern of immunogenic modulation was observed against a broad range of tumor-associated antigens, such as CEA, MUC1, PSA, and brachyury, and associated with augmented expression of multiple proteins involved in antigen processing and tumor immune recognition. Genetic and pharmacological inhibition studies identified HDAC1 as a key determinant in the reversal of carcinoma immune escape. Further, our findings suggest that the observed reversal of tumor immune evasion is driven by a response to cellular stress through activation of the unfolded protein response. This offers the rationale for combining HDAC inhibitors with immunotherapy, including therapeutic cancer vaccines.

## INTRODUCTION

Mounting evidence suggests that evasion of host immune surveillance is a key determinant of tumor progression [1-3]. Immune evasion is also a major obstacle to the efficacy of cancer immunotherapies, therefore preventing long-lasting tumor control. Hence, there is an unmet clinical need to develop effective therapeutic strategies to restore tumor immune recognition and promote long-lasting tumor control, which can be further augmented when combined with immunotherapy, such as immune checkpoint blockade or therapeutic cancer vaccines [4, 5].

Multiple strategies have been investigated to improve immune recognition of malignant tumors [6-8]. Recent evidence suggests that certain anticancer therapies can alter the biology of the surviving cell population to restore their sensitivity to T-cell-mediated lysis [6, 8, 9]. Mechanistic examination of this reversal of tumor immune evasion, also known as immunogenic modulation, determined it to be a consequence of a spectrum of biological adaptations to cellular stress, resulting in enhanced antigen processing and augmented tumor recognition [8-10]. Strong evidence has also implicated tumor epigenetic silencing of immune-associated genes as a determinant of an immune evasion signature [5, 11, 12]. Epigenetic deregulation has been associated with worse prognosis in a wide spectrum of malignancies, including in lung, breast and prostate [13-15]. Epigenetic silencing can occur at multiple levels, with DNA methylation and chromatin deacetylation having been identified as two major determinants [12, 16]. Unlike other types of malignant deregulation, such as oncogenic mutations, epigenetic alterations are mostly reversible, offering an exceptional therapeutic opportunity. However, despite its worth for the treatment of hematological malignancies, the promise of epigenetic therapy has not been realized for solid malignancies, albeit encouraging reports [4, 17]. Strong evidence from the last decade of clinical experience in the treatment of solid tumors with epigenetic agents strongly supports their use in combination with therapeutic modalities that can capitalize on the broad spectrum of tumor epigenetic reprogramming that they induce [4]. On this basis, multiple clinical studies have shown promising clinical activity in the management of solid malignancies when combining inhibitors of DNA methyltransferases (DNMT) or histone deacetylases (HDACs), including vorinostat and entinostat, with cytotoxic agents [4, 18].

Vorinostat is an orally bioavailable hydroxamate pan-HDAC inhibitor currently approved in the United States for the treatment of cutaneous T-cell lymphoma [13]. Vorinostat inhibits a broad spectrum of HDAC enzymes, namely class I (HDACs 1 to 3), and class IIb (HDACs 6 and 10), whereas entinostat specifically inhibits class I HDAC enzymes (HDACs 1 to 3, and 8) [13]. Both agents have shown synergistic antitumor activity in combination with checkpoint inhibitors and agonistic antibodies in murine models of solid malignancies [19, 20]. This synergy is in agreement with particular characteristics of these agents, including induction of immunogenic cell death by vorinostat, and suppression of tumor-initiating cells, regulatory T cells, and myeloid-derived suppressor cells by entinostat [21-23].

In a recent clinical report in which advanced stage, heavily pretreated non-small cell lung cancer (NSCLC) patients were treated with entinostat and the DNMT inhibitor azacitidine, 4 out of 19 patients showed major objective responses to subsequent anticancer therapies given immediately after epigenetic therapy, including immunotherapy targeting the checkpoint inhibitor PD1. Subsequent *in vitro* studies in NSCLC cell lines indicated that azacitidine induced an expression signature of immune genes and pathways [5], suggesting that epigenetic therapy of solid tumors may reprogram the tumor to reverse its immune evasion signature, thus priming it for a more efficient immune attack. This concept is further supported by *in vivo* and *in vitro* preclinical studies with HDAC inhibitors [22, 24]. However, findings on the effect of epigenetic modulation of immune genes in human carcinoma cell lines have been contradictory [25-27]. These discrepancies may be the result of tumor type inherent expression of specific HDAC enzymes as well as a consequence of very distinct and non-clinically observed drug overexposures used, potentially translating into a multitude of non-target effects.

Here we demonstrate that clinically relevant exposure to epigenetic therapeutic agents targeting HDAC1 reverses the immune evasion phenotype of prostate and breast carcinoma cells to antigen-specific lysis by cytotoxic T cells. Our data support a model of tumor immunogenic modulation where the reversal of epigenetic silencing promoting immune evasion is driven by a response to cellular stress through activation of the unfolded protein response (UPR). This offers the rationale for combining HDAC inhibitors with immunotherapy, including therapeutic cancer vaccines, in order to increase clinical benefit for patients harboring solid malignancies.

## RESULTS

### Vorinostat decreases pan-HDAC activity and proliferation of human carcinoma cells in an exposure-dependent manner

**Figure 1:**
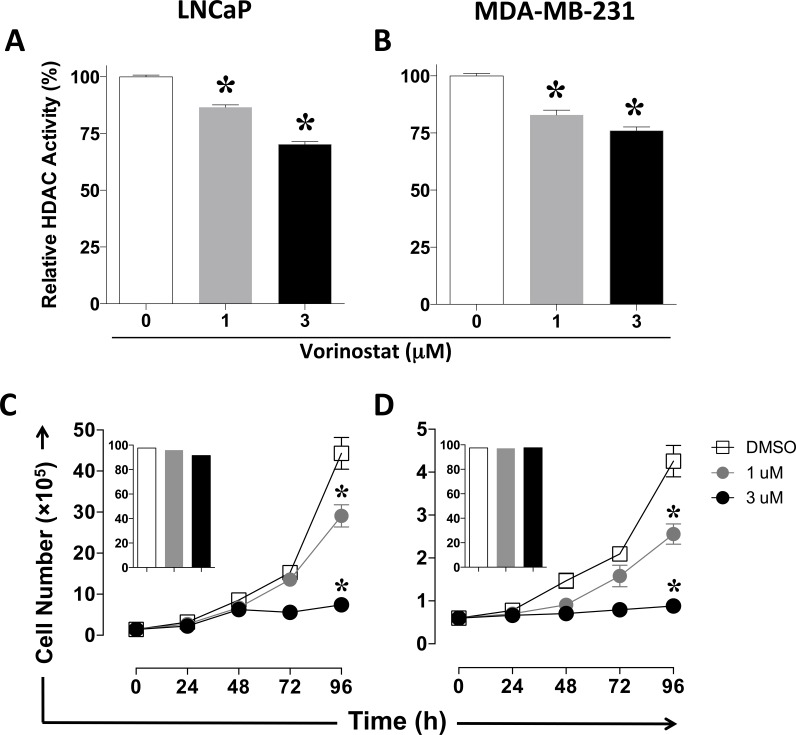
Vorinostat decreases pan-HDAC activity and proliferation of human carcinoma cells in an exposure-dependent manner Human prostate (LNCaP) and breast (MDA-MB-231) carcinoma cells were exposed to vorinostat (1 μM, grey circles and bars; 3 μM, black circles and bars), or vehicle (DMSO, open squares and bars). **A.** and **B.** HDAC activity determined at 96h. Results are presented as mean ± S.E.M. from replicate wells. **C.** and **D.** Cell number at the indicated time points. Insets denote viability at 96 h. Results are presented as mean ± S.D. from 6 replicate wells. Asterisks denote statistical significance relative to control cells exposed to vehicle (DMSO, *P* < 0.001). This experiment was repeated 2-3 times with similar results.

Supra clinical exposure of tumor cells to HDAC inhibitors, including vorinostat, has been shown to inhibit Class I and Class II histone deacetylases as well as exert antiproliferative effects [28, 29]. Thus, we first sought to examine *in vitro* the effect of clinically relevant exposure of human prostate (LNCaP) and breast (MDA-MB-231) carcinoma cells to vorinostat on the activity of HDAC enzymes (isoforms 1-11), cellular proliferation, and viability. Tumor cells were exposed daily for 5 h to 1 μM or 3 μM vorinostat, or vehicle (DMSO) over 4 consecutive days, mimicking the range of vorinostat exposure (Cmax, AUC) attained in cancer patients after oral once daily intake of 400 mg [30]. As shown in Figure 1, exposure to vorinostat significantly decreased HDAC activity in a dose-dependent manner in both prostate (Figure 1A, *P = 0.0006*) and breast (Figure 1B, *P = 0.0046*) carcinoma cells. In addition, significantly decreased cellular proliferation was also observed in a dose-dependent manner after exposure to vorinostat in both prostate (Figure 1C, *P < 0.0001*) and breast (Figure 1D, *P < 0.0001*) carcinoma cells relative to vehicle controls, with no significant effect observed on cellular viability (Figure 1C-1D insets). These data indicate that clinically relevant exposure of prostate and breast carcinomas to vorinostat induces target inhibition and promotes slower tumor growth. Vorinostat concentration of 3 μM was used for all subsequent experiments.

### Carcinoma cells exposed to vorinostat are significantly more sensitive to CTL-mediated killing

**Figure 2:**
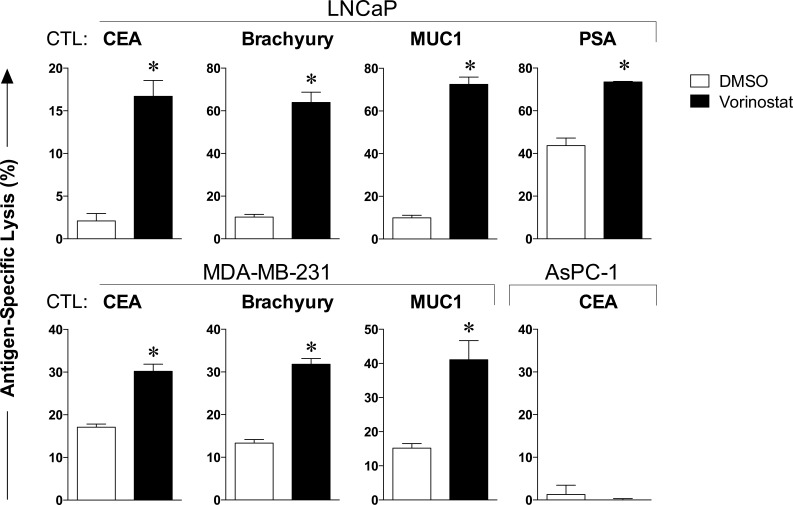
Carcinoma cells exposed to vorinostat are significantly more sensitive to CTL-mediated killing Human prostate (LNCaP) and breast (MDA-MB-231) carcinoma cells were exposed to vorinostat (3 μM, black bars) or to vehicle (DMSO, open bars) as described in Materials and Methods, prior to being used as targets for antigen-specific CTL lysis using CEA-, brachyury-, MUC1-, or PSA-specific CD8^+^ T cells as effector cells (E:T = 30:1). To verify that effector T cells were HLA-restricted, CTLs were incubated with HLA-A2 negative AsPC-1 pancreatic carcinoma cells exposed to vehicle (DMSO) or vorinostat. Results are presented as mean ± S.E.M. from 3-6 replicate wells, and are representative of 1-4 independent experiments. Asterisks denote statistical significance relative to controls.

We next examined the effect of clinically relevant vorinostat exposure on prostate and breast carcinoma cells' sensitivity to antigen-specific CTL-mediated lysis. LNCaP and MDA-MB-231 were exposed to vorinostat or to vehicle as before, prior to being used as targets for antigen-specific CTL lysis, using CEA-, brachyury-, MUC1-, or PSA-specific CD8^+^ T cells as effector cells. As shown in Figure 2, prostate carcinoma cells were significantly more sensitive to CTL-mediated lysis targeting CEA (*P = 0.002*), brachyury (*P = 0.0004*), MUC1 (*P < 0.0001*), or PSA (*P = 0.0011*). Similar results were observed with MDA-MB-231 breast carcinoma cells treated with vorinostat. The absence of significant lysis of HLA-A2 negative AsPC-1 pancreatic carcinoma cells exposed to vehicle or vorinostat confirmed that all effector T cells were HLA-A2 restricted. These data show that treatment of solid carcinomas with clinically relevant vorinostat exposures enhances antigen-specific CTL-mediated killing against a variety of tumor-associated antigens (TAAs) and across different tumor types, indicating a broad increase in tumor recognition by T cells.

### Vorinostat induces immunogenic modulation in carcinoma cells, including increased APM component expression

CTL killing of tumor targets requires T-cell recognition of specific major histocompatibility complex (MHC) Class I/CD8^+^-restricted epitope complexes on the surface of tumor cells, an event determined by the cooperative interactions of multiple antigen-processing machinery (APM) components. This suggests that the increased CTL-mediated lysis of tumor cells observed upon exposure to vorinostat may be a consequence of APM component upregulation. To test this hypothesis, MDA-MB-231 carcinoma cells were exposed to vorinostat or to vehicle as before. At the end of treatment, cells were examined by flow cytometry for intracellular expression of 6 APM components (Table 1). Exposure to vorinostat significantly increased the expression of 5 APM components by ≥ 25%, namely the immune proteosome subunits LMP2 and LMP7, the peptide transporter TAP1, the chaperone calnexin, and the HLA class I heavy chain-associated β2-microglobulin. Tapasin expression was also increased (22%) albeit to a lesser degree. We also observed increased expression of HLA class I antigens and ICAM-1, as well as the TAAs CEA and MUC1 on the surface of tumor cells upon exposure to vorinostat. These data indicate that HDAC inhibition upregulates multiple APM components; this change is likely to enhance the synthesis and expression of HLA class I antigen-TAA derived peptide complexes, resulting in increased T-cell recognition and lysis of tumor targets exposed to vorinostat.

**Table 1:** Effect of vorinostat on protein expression of APM components in human breast carcinoma cells

			% Positive (MFI)	
			DMSO	Vorinostat
Antigen Processing Machinery	Intracellular	LMP2	86 (251)	95 (**363**)
		LMP7	19 (180)	14 (190)
		TAP1	43 (153)	**59** (171)
		Tapasin	64 (136)	78 (160)
		Calnexin	85 (560)	98 (**857**)
		β2-microglobulin	71 (115)	**96 (144)**
	Extracellular	HLA-ABC	99 (195)	100 (**307**)
		HLA-A2	99 (303)	99 (304)
		ICAM-1	77 (46)	**95 (131)**
		CEA	14 (40)	**21** (23)
		MUC1	9 (14)	**29 (24)**

MDA-MB-231 cells were exposed to vorinostat (3 μM) or vehicle (DMSO) control. At the end of treatment (96h), cells were analyzed by flow cytometry for cellular expression of indicated APM components. Bold denotes significant modulation (≥ 25% change in percent of cells or MFI not observed in isotype control vs. untreated cells).

### Vorinostat-induced immunogenic modulation of MDA-MB-231 carcinoma cells is mediated by HDAC1

**Figure 3:**
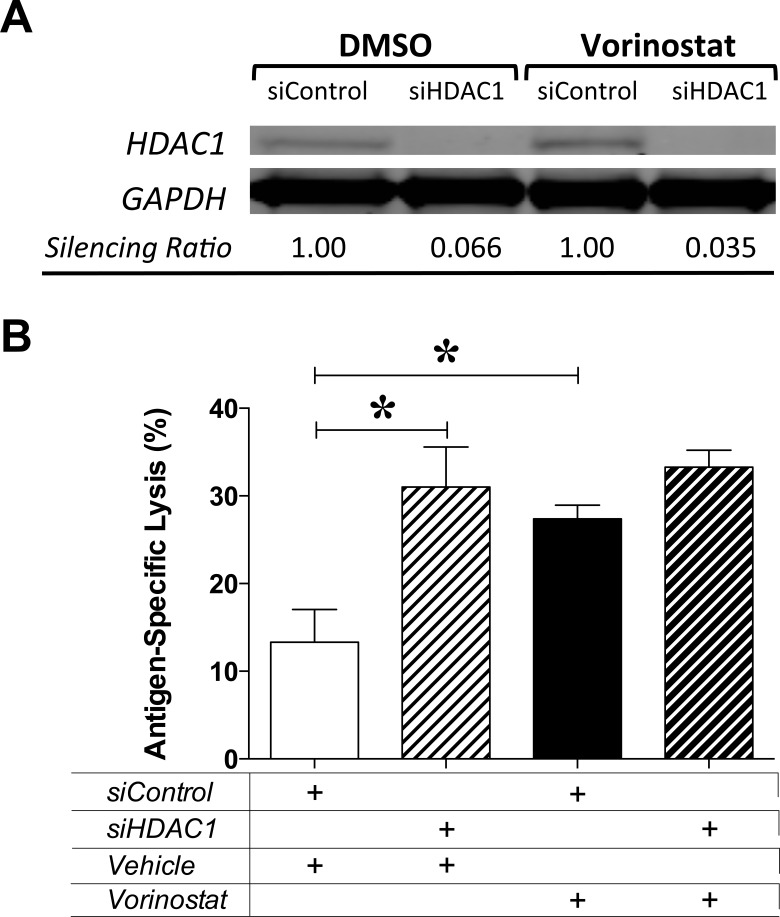
Vorinostat-induced immunogenic modulation of MDA-MB-231 carcinoma cells is mediated by HDAC1 MDA-MB-231 cells were exposed to siRNA control or targeting HDAC1 for 24 h prior to being exposed to vehicle (DMSO) or vorinostat (3 μM) as described in Materials and Methods. **A.** At the end of treatment, total cell lysates were examined by Western blotting to determine expression of HDAC1. GAPDH was used as internal control for total protein levels. Silencing ratio denotes HDAC1 protein expression relative to GAPDH, further normalized to protein levels after treatment in the presence of silencing RNA control. **B.** At the end of treatment, MDA-MB-231 cells were used as targets in a CTL-lysis assay where effector brachyury-specific CD8^+^ T cells were used at an E:T ratio of 30:1. Results are presented as mean ± S.E.M. from 4-6 replicate wells. Asterisks denote statistical significance relative to controls (*P = 0.002). Data is representative of two independent experiments.

Class I HDAC1-3 are major targets of vorinostat, and have been shown to be co-repressors of gene transcription, including genes involved in tumor immune recognition [13, 31, 32]. We hypothesized that this class of HDACs mediates vorinostat-induced immunogenic modulation of tumor cells, thus rendering them more sensitive to CTL-mediated killing. To test this hypothesis, MDA-MB-231 cells were exposed to silencing RNA (siRNA) targeting HDAC1 or control siRNA for 24h prior to exposure to vehicle or vorinostat as before. As shown in Figure 3A, HDAC1 expression in tumor targets treated with siRNA targeting HDAC1 was significantly decreased at the end of treatment compared with targets exposed to control siRNA. At the end of treatment, tumor cells were used as targets for brachyury-specific T-cell-mediated lysis. As shown in Figure 3B, vorinostat exposure significantly augmented CTL sensitivity of MDA-MB-231 target cells exposed to control siRNA, a 2-fold increase relative to vehicle controls (*P = 0.0024*). Strikingly, the augmented CTL lysis attained in silencing control targets after exposure to vorinostat was also observed upon silencing of HDAC1 in the absence of vorinostat exposure. Moreover, treatment of HDAC1-silenced MDA-MB-231 tumor cells with vorinostat did not further increase CTL lysis relative to vehicle control. Altogether, this data suggest that vorinostat-induced immunogenic modulation of MDA-MB-231 breast carcinoma cells is mediated by HDAC1.

### HDAC inhibition activates the ER stress responsive element in an exposure-dependent manner

**Figure 4:**
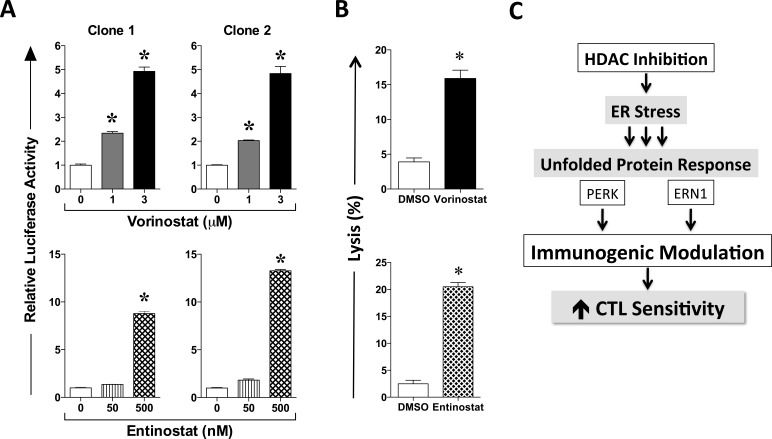
HDAC inhibition activates the ER stress responsive element in LNCaP carcinoma cells in a dose-dependent manner **A.** Single-cell clones of LNCaP cells stably transduced with an ER stress responsive element driving firefly luciferase expression were exposed to vorinostat or entinostat, at the designated concentrations, or DMSO controls, as described in Materials and Methods. At the end of treatment, firefly and renilla luciferase activities were determined. Data are shown as the ratio of firefly luciferase activity relative to that of control renilla luciferase within each well, further normalized to DMSO control. Results are presented as mean ± S.E.M. from 4-6 replicate wells, and are representative of two independent experiments. **B.** Parental LNCaP prostate carcinoma cells were exposed to vorinostat (3 μM), entinostat (500 nM) or to vehicle (DMSO) controls as described in Materials and Methods, prior to being used as targets for antigen-specific CTL lysis using PSA-specific CD8^+^ T cells as effector cells (E:T = 30:1). Results are presented as mean ± S.E.M. from 6 replicate wells. Asterisks denote statistical significance relative to controls (*P* < 0.05). **C.** Schematic representation of immunogenic modulation induced by HDAC inhibition in human carcinoma cells.

We have previously demonstrated that immunogenic modulation and augmented immune recognition of human carcinoma cells by cognate cytotoxic T cells encompasses a tumor adaptive response to endoplasmic reticulum stress through the UPR [10]. HDAC1, a Class I HDAC and main vorinostat target, has been shown to control the transcription of Grp78, an ER stress responsive gene by directly binding to the ERSE [33]. We hypothesized that vorinostat may therefore activate the ER stress response through HDAC1 inhibition. To test this hypothesis, two single-cell clones of LNCaP cells stably transduced with an ERSE reporter driving firefly luciferase expression were exposed to vorinostat or vehicle as before. As shown in Figure 4A, vorinostat activated ERSE in a dose-dependent manner. To further examine the induction of ER stress through Class I HDAC inhibition, ERSE reporter clones were treated with clinically relevant exposures of entinostat, a selective Class I HDAC inhibitor [13]. Similarly to vorinostat, tumor exposure to entinostat activated ERSE in an exposure-dependent manner (Figure 4A), resulting in increased sensitivity to CTL-mediated killing similar to that with vorinostat (Figure 4B). Altogether, this data indicates that HDAC inhibition with agents targeting Class I HDAC enzymes induces ER stress, which ultimately results in immunogenic modulation and increased tumor sensitivity to CTL-mediated lysis (Figure 4C).

### The unfolded protein response mediates vorinostat-induced immunogenic modulation

**Figure 5:**
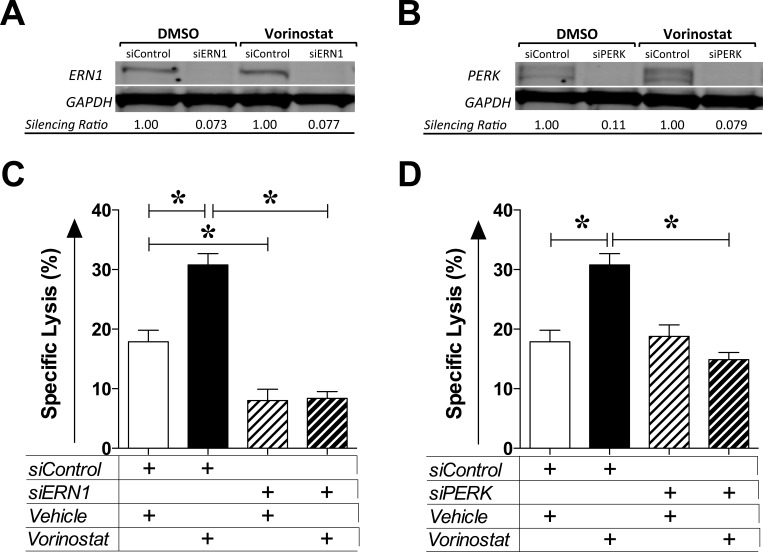
Vorinostat-induced immunogenic modulation is mediated by the unfolded protein response MDA-MB-231 cells were exposed to siRNA control or targeting ERN1 or PERK for 24 h prior to being exposed to vehicle (DMSO) or vorinostat (3 μM) as described in Materials and Methods. **A.** and **B.** At the end of treatment, total cell lysates were examined by Western blotting to determine expression of ERN1 (A) or PERK (B). GAPDH was used as internal control for total protein levels. Silencing ratio denotes target protein expression relative to GAPDH, further normalized to protein levels after treatment in the presence of silencing RNA control. **C.** and **D.** At the end of treatment, MDA-MB-231 cells were used as targets in a CTL lysis assay using CEA-specific CD8^+^ T cells as effectors (E:T = 30:1). Results are presented as mean ± S.E.M. from 6 replicate wells, and are representative of 2-3 independent experiments. Asterisks denote statistical significance relative to controls (*P* < 0.0001).

ER stress activates the UPR, an adaptive reaction attempting to restore ER homeostasis through a cascade of cellular events [34]. To examine the functional consequence of ER stress induced by HDAC inhibition and the possible involvement of the UPR, MDA-MB-231 cells were exposed to siRNA control or targeting two independent ER stress/UPR sensors, ERN1 or PERK, for 24 h prior to being exposed to vehicle or vorinostat as before. At the end of treatment, gene silencing was confirmed (Figure 5A-5B) and tumor cells were used as targets for CEA-specific CTL lysis (Figure 5C-5D). As shown in Figure 5C, exposing MDA-MB-231 cells to control siRNA led to significantly increased target lysis by cytotoxic T cells following vorinostat treatment (*P < 0.0001*). However, vorinostat did not increase CTL lysis of tumor cells when ERN1 (Figure 5C) or PERK (Figure 5D) were silenced. Collectively, these data suggest that the increased sensitivity of human carcinoma cells to CTL-mediated lysis as a result of HDAC inhibition stems from a cellular survival response to ER stress mediated through the UPR.

## DISCUSSION

Malignant tumors can evade host immune surveillance through multiple mechanisms, including epigenetic silencing of genes involved in immune recognition [1, 11, 22]. Innovative therapies, including epigenetic reprogramming, are being actively examined to restore tumor immune recognition for better tumor control [1, 7, 8, 10, 35]. Epigenetic therapies have shown variable levels of clinical benefit for patients harboring solid tumors, either as monotherapy or in combination with chemotherapy [4]. In a phase I/II trial where heavily pretreated NSCLC patients received combined epigenetic therapy with azacitidine and entinostat, inhibitors of DNA methylation and histone deacetylation, respectively, objective responses were observed, including a complete response and a durable partial response [17]. However, the most striking observation in this study was that 4 out of 19 patients had major objective responses to subsequent anticancer therapies given immediately after epigenetic therapy. Two out of the 4 patients were treated with monoclonal antibodies targeting vascular endothelial growth factor (VEGF) or programmed cell death protein-1 (PD-1). Subsequent *in vitro* studies from the same group in NSCLC cell lines showed that azacitidine induced an expression signature of immune genes and pathways [5]. However, the effect of entinostat was not reported. These observations highlight the potential clinical benefit of “epigenetic priming” in the treatment of solid carcinomas, where epigenetic therapy reprograms the tumor for subsequent response to immunotherapy, including immune checkpoint blockade and, potentially, therapeutic cancer vaccines. These findings prompted the design of several clinical interventions for the treatment of solid tumors, combining epigenetic therapy with immunotherapeutics targeting Her2, PD-1, and CTL-associated antigen 4 (CTLA-4). However, the combination of epigenetic therapy and vaccine immunotherapy has not been examined clinically.

Here we demonstrate that LNCaP and MDA-MB-231 carcinoma cells are more sensitive to T-cell-mediated lysis *in vitro* after clinically relevant exposure to epigenetic therapy with either the pan-HDAC inhibitor vorinostat (Figure 2) or the class I HDAC inhibitor entinostat (Figure 4). This increased immune recognition was observed against very distinct HLA class I/epitope complexes, specific for a broad range of TAA, such as CEA, MUC1, PSA, and brachyury. Similar results were observed with additional cell lines representative of distinct tumor types, including breast (MCF-7; ER+), and colon (SW620, SW480) carcinomas (data not shown).

This pattern of increased sensitivity to antigen-specific CTL lysis suggests that HDAC inhibition induces immunogenic modulation by promoting a signature of immune recognition across multiple solid carcinoma types. This is of particular importance, as several TAAs, HLA class I family of proteins, APM components, and costimulatory molecules have been shown to be epigenetically silenced or downregulated in malignancies of diverse origin, hampering tumor immune recognition by cognate cytotoxic T cells and contributing to poor prognosis, including in breast and prostate cancer [1, 2, 11, 36, 37]. However, supra clinical exposure of human carcinoma cells to vorinostat has previously been shown to result in upregulation of HLA related genes [31, 35]. Here we demonstrate that clinically translatable exposure of carcinoma cells to vorinostat reprograms multiple elements of the APM machinery, thereby augmenting tumor recognition and lysis by cytotoxic T cells (Table 1).

The anti-proliferative effect of HDAC inhibition in breast and prostate carcinoma cells reported here (Figure 1) is a common feature of HDAC1/2 inhibition [38] that has been observed preclinically in other tumor types [29, 39], as well as in breast cancer patients treated with vorinostat [40]. Class I HDAC-1 to -3 are major targets of both vorinostat and entinostat, and have been shown to be co-repressors of gene transcription, including selected genes involved in tumor immune recognition [13, 31, 32]. Here we demonstrate that immunogenic modulation promoted by these HDAC inhibitors is a consequence of direct target inhibition, as silencing HDAC1 in tumor targets increases their sensitivity to CTL killing to the same extent as pharmacological inhibition with vorinostat, with no additive effect of vorinostat observed in targets with silenced HDAC1 (Figure 3). Our data highlight a novel role for class I HDAC inhibitors in the epigenetic reprogramming of solid carcinomas through on-target reversal of T-cell immune evasion.

We have previously demonstrated that immunogenic modulation and augmented immune recognition by cytotoxic T cells of human carcinomas surviving radiation exposure encompass a tumor adaptive response to endoplasmic reticulum stress through UPR activation [9, 10]. Interestingly, HDAC1 has been shown to repress the activity of several transcription factors that control the expression of ER stress responsive genes by directly binding and activating the ERSE [33]. Both vorinostat and entinostat activate the ERSE in an exposure-dependent manner and both render prostate and breast (not shown) carcinoma cells more amenable to CTL-mediated lysis (Figure 4). Our findings suggest that HDAC inhibition with agents targeting Class I HDAC enzymes induces ER stress, which ultimately results in immunogenic modulation and increased tumor sensitivity to CTL-mediated lysis (Figure 4C). This is further supported by our findings demonstrating an essential role for ERN1 and PERK in vorinostat-induced immunogenic modulation (Figure 5), two distinct endoplasmic reticulum stress sensors and mediators of the UPR.

The findings here provide a mechanistic rationale for hypothesis-driven clinical studies for patients with solid carcinomas treated with class I HDAC inhibitors followed by or in combination with immunotherapy, particularly therapeutic cancer vaccines, where reversal of a tumor immune evasion signature into a more indolent, slower growing, tumor phenotype may translate to heightened clinical benefit [41, 42].

## MATERIALS AND METHODS

### Tumor-cell lines

Human carcinoma cells of breast [MDA-MB-231 (ATCC^®^ HTB-26^™^)], prostate [LNCaP clone FGC (ATCC^®^ CRL-1740^™^)], and pancreas [AsPC-1 (ATCC^®^ CRL-1682^™^)] origin were obtained from American Type Culture Collection (ATCC) and cultured in medium designated by the provider for propagation and maintenance. All cell lines were used at low passage number and proven free of *Mycoplasma*.

### Chemicals and drug exposure

Vorinostat and entinostat were obtained from Selleck Chemicals. Adherent tumor cells in log-growth phase were exposed daily to vehicle (DMSO) or vorinostat at the indicated concentrations for 5 h, over 4 consecutive days. At the end of each treatment, cells were washed in fresh medium and returned to incubation at 37°C with 5% CO_2_. Alternatively, cells were continuously exposed to vehicle (DMSO) or entinostat at the indicated concentrations for 72 h.

### Analysis of cell growth and viability

Tumor cells were exposed to DMSO or vorinostat as described above. Cells were harvested daily and viable cells were counted by trypan blue exclusion using a Cellometer Auto T4 automated cell counter (Nexcelom Bioscience). Cellular viability was confirmed by flow cytometry using Live/Dead exclusion, according to manufacturer's instructions (Invitrogen).

### HDAC activity assay

Changes in the nuclear enzyme activity of HDAC isoforms 1-11 following vorinostat treatment of MDA-MB-231 and LNCaP cells were determined using the colorimetric EpiQuik HDAC Activity/Inhibition Assay Kit (Epigentek). Briefly, 10 μg of extracted nuclear HDAC proteins were incubated with acetylated HDAC substrate for 90 min at 37°C. HDAC deacetylated products were detected following sequential incubation with capture and detection antibodies, according to the manufacturer's specifications.

### CD8^+^ cytotoxic T-cell (CTL) lines

Carcinoembryonic antigen (CEA)-specific CTLs recognize the CEA peptide epitope YLSGANLNL (CAP-1) [43]. Prostate-specific antigen (PSA)-specific CTLs recognize the PSA peptide epitope VLSNDVCAQV [44]. The mucin-1 (MUC1)-specific CD8^+^ CTL line, designated MUC1 CTL, recognizes the MUC1 peptide epitope ALWGQDVTSV [45]. Brachyury-specific CTLs recognize the brachyury peptide epitope WLLPGTSTL (T-p2) [46]. All T-cell lines were HLA-A2-restricted.

### Cytotoxicity assays

Carcinoma cells exposed to vorinostat, entinostat, or vehicle (DMSO) were labeled with ^111^In prior to being used as targets for direct lysis by effector CTLs at an effector-to-target ratio of 30:1 in a standard overnight cytotoxicity ^111^In-release assay [9].

### Gene silencing and western blots

Silencer^®^ siRNA and negative control siRNA were used to silence HDAC1, ERN1, and PERK in MDA-MB-231 carcinoma cells, according to the manufacturer's instructions (Life Technologies). Cells were exposed to siRNA 24 h prior to treatment with vorinostat or DMSO for 4 consecutive days, as described above. At the end of treatment, cells were harvested and used as CTL targets. The expression level of targeted proteins was examined by Western blotting of cell lysates prepared in RIPA buffer containing 1 mM PMSF (Cell Signaling Technology). Proteins (20-40 μg) were separated using 4%-12% MOPS SDS-PAGE (Life Technologies) then transferred to nitrocellulose membranes. Primary antibodies specific for HDAC1, ERN1, PERK, and GAPDH were acquired from Cell Signaling Technology. Blots were incubated with anti-rabbit IRDye secondary antibodies (LI-COR Biotechnology). Detection and quantification of band intensity were performed with the Odyssey Infrared Imaging System (LI-COR Biotechnology). Protein levels were normalized to the loading control GAPDH.

### Luciferase ER stress reporter assays

Human prostate carcinoma LNCaP cells were stably transduced with replicant-incompetent lentiviral particles expressing an inducible reporter construct encoding the firefly luciferase gene under the control of a basal promoter element (TATA box) joined to tandem repeats of the endoplasmic reticulum (ER) stress transcriptional response element (ERSE) (Qiagen). As an internal control, cells were co-transduced with lentiviral particles expressing a constitutive Renilla luciferase expression cassette under the control of the CMV promoter (Qiagen). Transduced cells were selected in media containing 1 μg/ml puromycin (Life Technologies) and single-cell clones were selected for study. Luciferase activity was quantified using the Dual-Luciferase Reporter Assay (Promega).

### Flow cytometry analysis

Cell-surface and intracytoplasmic staining was performed as previously described [47]. Surface staining of tumor cells was performed using the primary labeled monoclonal antibodies HLA-A2-FITC, ICAM-1 (CD54)-PE, CEA (CD66)-FITC, MUC1 (CD227)-FITC, and the appropriate isotype-matched controls (BD Biosciences). For intracellular analysis of antigen processing machinery (APM) components, mouse IgG1 (MK2-23) isotype control, LMP2 (SY-1)-, LMP7 (HB2)-, TAP-1 (NOB1), calnexin (TO-5)-, β2-microglobulin (L368), and tapasin (TO-3)-specific monoclonal antibodies were developed and characterized as described [48-50]. Cellular fluorescence of 3×10^4^ cells was examined on a FACSCalibur flow cytometer using CellQuest software (BD Biosciences). Proteins were scored as markedly upregulated if confirmed statistically and if detection levels and/or mean fluorescence intensity (MFI) increased ≥ 25% following treatment and were not observed in control cells vs. isotype controls.

### Statistical analysis

The effect of repetitive drug exposure over time on cellular proliferation was examined by 2-way ANOVA. Significant differences between multiple treatment groups were determined by 1-way ANOVA with Tukey's comparison, both based on a confidence interval of 95% using Prism 6.0f software (GraphPad Software Inc.). Alternatively, statistical differences between 2 treatments were analyzed by unpaired Student's t test with a 2-tailed distribution, unless reported otherwise, and reported as P values. Significant differences in the distribution of flow cytometry analysis data were determined by the Kolmogorov-Smirnov test using CellQuest software (BD Biosciences).

## Abbreviations

APM: antigen processing machinery
CEA: carcinoembryonic antigen
CTL: cytotoxic T-cell
CTLA-4: CTL-associated antigen 4
DNMT: DNA methyltransferase
ER: endoplasmic reticulum
ERN1: Endoplasmic Reticulum To Nucleus Signaling 1
ERSE: ER stress response element
HDAC: histone deacetylase
MFI: mean fluorescence intensity
MHC: major histocompatibility complex
MUC1: mucin-1
NSCLC: non-small cell lung cancer
siRNA: silencing RNA
PD-1: programmed cell death protein-1
PERK: protein kinase R (PKR)-like endoplasmic reticulum kinase
PSA: prostate-specific antigen
UPR: unfolded protein response
TAA: tumor-associated antigen
VEGF: vascular endothelial growth factor.
